# Successful One-Stage Revision With Continuous Local Antibiotic Perfusion (CLAP) for Delayed Periprosthetic Joint Infection After Reverse Shoulder Arthroplasty: A Case Report

**DOI:** 10.7759/cureus.100273

**Published:** 2025-12-28

**Authors:** Jin Nagasawa, Yoshihiro Hirakawa, Katsumasa Nakazawa, Yoichi Ito, Tomoya Manaka

**Affiliations:** 1 Orthopaedic Surgery, Ishikiriseiki Hospital, Higashiōsaka, JPN; 2 Orthopaedic Surgery, Osaka Metropolitan University Graduate School of Medicine, Osaka, JPN; 3 Orthopaedic Surgery, Ito Clinic, Osaka Shoulder Center, Matsubara, JPN

**Keywords:** clap, multidrug-resistant organism (mdro), one-stage revision, periprosthetic joint infection (pji), reverse shoulder arthroplasty (rsa)

## Abstract

Periprosthetic joint infection (PJI) is a serious complication following reverse shoulder arthroplasty (RSA). We report a case of delayed PJI after RSA that was successfully treated with one-stage revision surgery combined with continuous local antibiotic perfusion (CLAP). A 72-year-old man with diabetes underwent RSA for cuff tear arthropathy at another hospital 21 months ago. Four months before presentation, he developed pain, swelling, warmth, and discharge from the surgical site, and PJI was diagnosed. Initial debridement, implant exchange, and antibiotic therapy were attempted at the previous hospital; however, the infection persisted, prompting a referral to our institution. On admission, wound drainage was observed; blood tests showed WBC 6,400/μL and C-reactive protein (CRP) 0.07 mg/dL. Culture identified extended-spectrum β-lactamase (ESBL)-producing *Klebsiella oxytoca* and *Staphylococcus aureus*. Plain radiography revealed osteolysis around both the glenoid and humeral components. A diagnosis of chronic PJI was made, and the patient underwent one-stage revision surgery with debridement, implant exchange, and CLAP therapy (gentamicin). Postoperative management included two weeks of negative pressure wound therapy (NPWT) with continuous gentamicin perfusion, followed by six weeks of intravenous antibiotics. Infection signs resolved rapidly, and at one year postoperatively, the patient showed no recurrence of infection without antibiotics. The active range of motion of the right shoulder at one year was as follows: forward flexion, 100°; abduction, 90°; external rotation, 10°; internal rotation to the sacrum; and JOA score, 69. This case highlights the potential role of CLAP as an adjunct to one-stage revision for shoulder PJI, even in infections caused by multidrug-resistant organisms.

## Introduction

Periprosthetic joint infection (PJI) after reverse shoulder arthroplasty (RSA) is a serious complication that is difficult to eradicate because of biofilm formation and indolent pathogens. For chronic shoulder PJI, debridement, antibiotics, and implant retention are usually insufficient, and most guidelines and treatment algorithms recommend prosthesis exchange, most commonly as a two-stage revision with an antibiotic spacer [1,2]. However, two-stage revision requires multiple operations and prolonged treatment, can damage or scar soft tissues, delay rehabilitation, and place a considerable physical and economic burden on patients [2,3].

In response to these limitations, one-stage exchange has gained attention as a less invasive option for carefully selected patients. Hip and knee studies report that, when the causative organisms are identified, soft tissue and bone stock are adequate, and targeted antimicrobial therapy is available, one-stage revision can provide infection control and functional outcomes comparable to two-stage strategies while reducing treatment burden [4-6]. Similar concepts have been applied to the shoulder, where selected chronic PJI cases have been managed with one-stage revision combined with meticulous debridement and appropriate antimicrobial therapy while preserving the function [2,7].

Continuous local antibiotic perfusion (CLAP) is a local antibiotic delivery technique developed in Japan that maintains sustained high antibiotic concentrations at the infection site while limiting systemic exposure, and has shown promising results in refractory musculoskeletal and periprosthetic infections [8,9]. This approach is particularly useful for infections caused by multidrug-resistant organisms such as extended-spectrum β-lactamase (ESBL)-producing gram-negative bacteria, which restrict systemic β-lactam options but often remain susceptible to aminoglycosides, because gentamicin-based CLAP can provide intensive local exposure to an active agent during and after revision surgery. Here, we report a case of chronic shoulder PJI after RSA caused by ESBL-producing *Klebsiella oxytoca* and *Staphylococcus aureus*, which was successfully treated with one-stage revision combined with CLAP.

## Case presentation

A 72-year-old right-handed man with diabetes mellitus (HbA1c 6.7%) underwent RSA for cuff tear arthropathy of the right shoulder at a different institution. Twenty-one months after the primary surgery, he developed increasing shoulder pain, swelling, redness, and purulent discharge from the operative site. A fistula had formed, which was diagnosed as an infection by the previous doctor, and debridement with insert exchange was performed, followed by systemic antibiotics. According to the referral documents, modular components were exchanged approximately one month before presentation to our hospital; however, detailed information on which specific components were replaced, the exact systemic antibiotic regimens, and intraoperative culture results at the referring hospital were not available. Infection control remained unsuccessful, and he was therefore referred to our institution because the symptoms had not improved even after one month had passed since the surgery.

**Figure 1:**
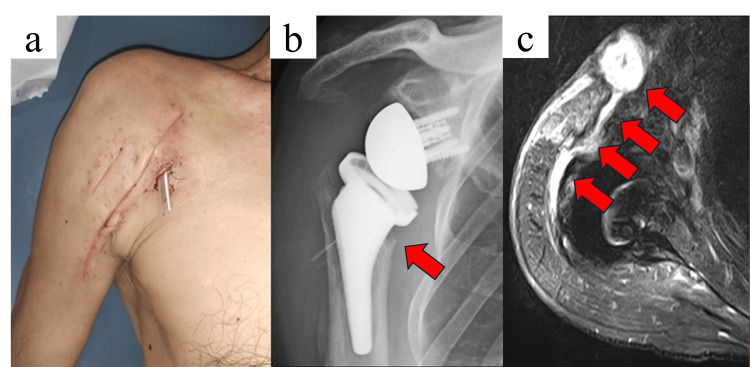
Preoperative findings of the right shoulder. a. Clinical photograph showing erythema, swelling, and purulent discharge with a sinus tract at the previous surgical site. b. Anteroposterior radiograph demonstrating osteolysis around the humeral component following reverse shoulder arthroplasty. c. Axial STIR magnetic resonance image revealing fluid collection and fistula formation changes suggestive of PJI. STIR: short tau inversion recovery; PJI: periprosthetic joint infection

Upon admission, the patient was afebrile but exhibited a sinus tract with continuous purulent drainage (Figure 1a). Laboratory studies showed a mild increase in inflammatory response (Table 1). Joint aspiration revealed ESBL-producing *K. oxytoca* and *S. aureus*, both retaining susceptibility to aminoglycosides. Antimicrobial susceptibility testing showed that the ESBL *K. oxytoca* isolate was resistant to third-generation cephalosporins but remained susceptible to carbapenems and aminoglycosides (including gentamicin). The *S. aureus* isolate was susceptible to standard anti-staphylococcal agents and gentamicin. These susceptibility profiles support the use of carbapenem-based systemic therapy in combination with local high-concentration gentamicin perfusion. Radiographs demonstrated osteolysis around the humeral component (Figure 1b), and MRI revealed fluid collection and fistula formation suggestive of PJI (Figure 1c).

Laboratory studies showed a mild inflammatory response (Table 1).

**Table 1:** Patient laboratory data WBC: white blood cell; CRP: C-reactive protien; ESR-1H: erythrocyte sedimentation rate, one hour

Parameter	Patient Value	Reference Range
WBC	6400	3500-9000/µL
CRP	0.07	≦0.3 mg/dL
Neutrophil count	74.50%	40-75%
ESR-1H	38	2-10 mm/hour

Based on the ICM 2018 Shoulder PJI Criteria [1], the presence of a sinus tract and positive cultures of the same pathogens in multiple samples fulfilled the major criteria for definite PJI. Considering the identified pathogens, preserved soft-tissue envelope, and antibiotic susceptibilities, a one-stage revision arthroplasty combined with CLAP was selected.

**Figure 2:**
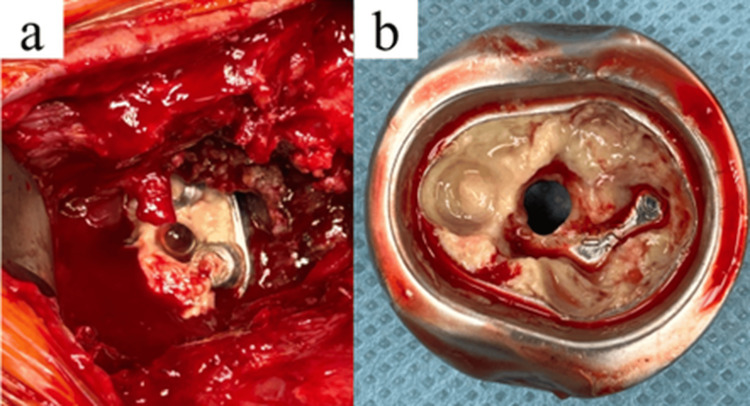
Intraoperative findings during one-stage revision surgery a. Intraoperative view showing purulent tissue around the baseplate, consistent with periprosthetic joint infection. b. Retrieved humeral component covered with necrotic tissue on the inner surface.

During surgery, purulent and necrotic tissues were observed around the humeral stem and the glenoid baseplate (Figure 2). All reverse shoulder components, including the cementless humeral stem, glenoid baseplate, glenosphere, and polyethylene liner, were removed.

After implant removal and extensive debridement, the site was irrigated with 6 L of saline containing diluted povidone-iodine and gentamicin. The glenoid and humeral sides were exchanged using new components after confirming adequate bone stock and viable soft tissue. The operative time was 157 minutes, with an estimated blood loss of 600 mL. The primary implant was a cementless Exactech short stem. During revision, the humeral and glenoid sides were exchanged using the Equinoxe Reverse Shoulder System (Exactech, Inc., Gainesville, Florida, United States) with a press-fit standard humeral stem (size 9) and a long-post glenoid baseplate (+10 mm).

**Figure 3:**
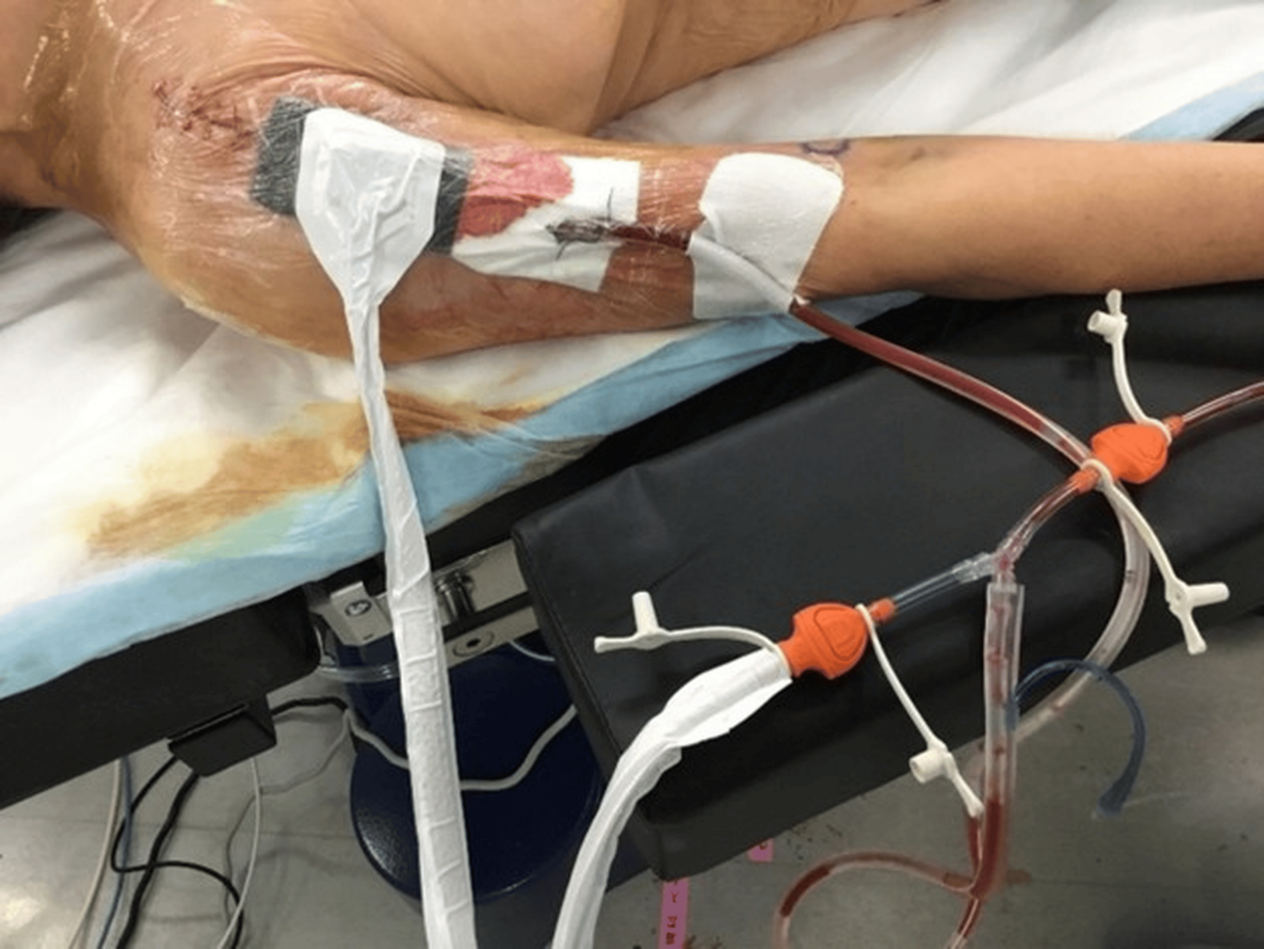
Postoperative management with continuous local antibiotic perfusion (CLAP). A negative pressure wound therapy (NPWT) system combined with continuous local antibiotic perfusion using gentamicin (60 mg/50 mL at 2 mL/h) was applied. NPWT was set to continuous negative pressure, and the dressing was changed every two to three days. The inflow catheter was inserted into the glenohumeral joint space, and the outflow catheter was incorporated into the NPWT foam dressing. The catheters and tubing were secured with sutures and an additional adhesive drape to prevent leaks and maintain a closed circuit, increasing local antibiotic concentrations at the surgical site while minimizing systemic exposure.

After revision, CLAP therapy was initiated using gentamicin (60 mg/50 mL; 1,200 μg/mL) continuously infused at 2 mL/hour through a dual-catheter system integrated with negative pressure wound therapy (NPWT) (Figure 3). The inflow catheter was placed in the joint cavity, and the outflow catheter into the NPWT sponge, creating a closed circulation circuit to maintain high local antibiotic concentration while minimizing systemic exposure.

**Figure 4:**
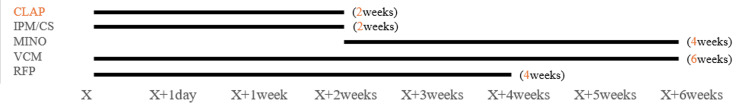
Postoperative CLAP and antibiotic treatment duration. After revision, CLAP and antibiotic treatment were initiated. The treatment periods are shown. CLAP: continuous local antibiotic perfusion; VCM: vancomycin; MINO: minocycline; RFP: rifampicin; IPM/CS: imipenem/cilastatin.

CLAP therapy was continued for 14 days, followed by six weeks of intravenous(imipenem/cilastatin: 0.5 g × 2/day, vancomycin: 0.5 g × 2/day) and oral (minocycline: 100 mg × 2/day, rifampicin: 150 mg × 3/day) antibiotic therapy. In accordance with CLAP management recommendations, serum gentamicin trough levels were monitored for two to three days after initiation and then twice weekly, aiming to maintain trough values at or below 1 μg/mL, and renal function was checked in parallel (Figure 4). The postoperative course was uneventful, with gradual wound healing and normalization of inflammatory markers (Table 2). The results of the intraoperative culture test also revealed bacteria similar to those detected in the preoperative arthrocentesis results.

**Table 2:** Postoperative clinical course following one-stage revision with CLAP therapy. Inflammatory markers remained within normal range throughout the follow-up period, indicating sustained infection control after single-stage reconstruction with continuous local antibiotic irrigation (CLAP). Renal function also remained stable. CRP: C-reactive protein; BUN: blood urea nitrogen;

	Day X	X+1 day	X+1 week	X+2 weeks	X+3 weeks	X+4 weeks	X+5 weeks	X+6 weeks	X+7 weeks	X+1 year
WBC (×10² /µL)	6400	5000	5500	3900	3400	3500	4800	6100	4500	7600
CRP (mg/dL)	0.07	1.3	0.31	0.02	0.17	0.21	0.12	0.01	0.02	0.01
BUN (mg/dL)	17	15	10	11	23	20	12	16	14	21
Creatinine (mg/dL)	1.09	0.48	0.73	0.61	0.75	0.71	0.64	0.72	0.73	0.83

**Figure 5:**
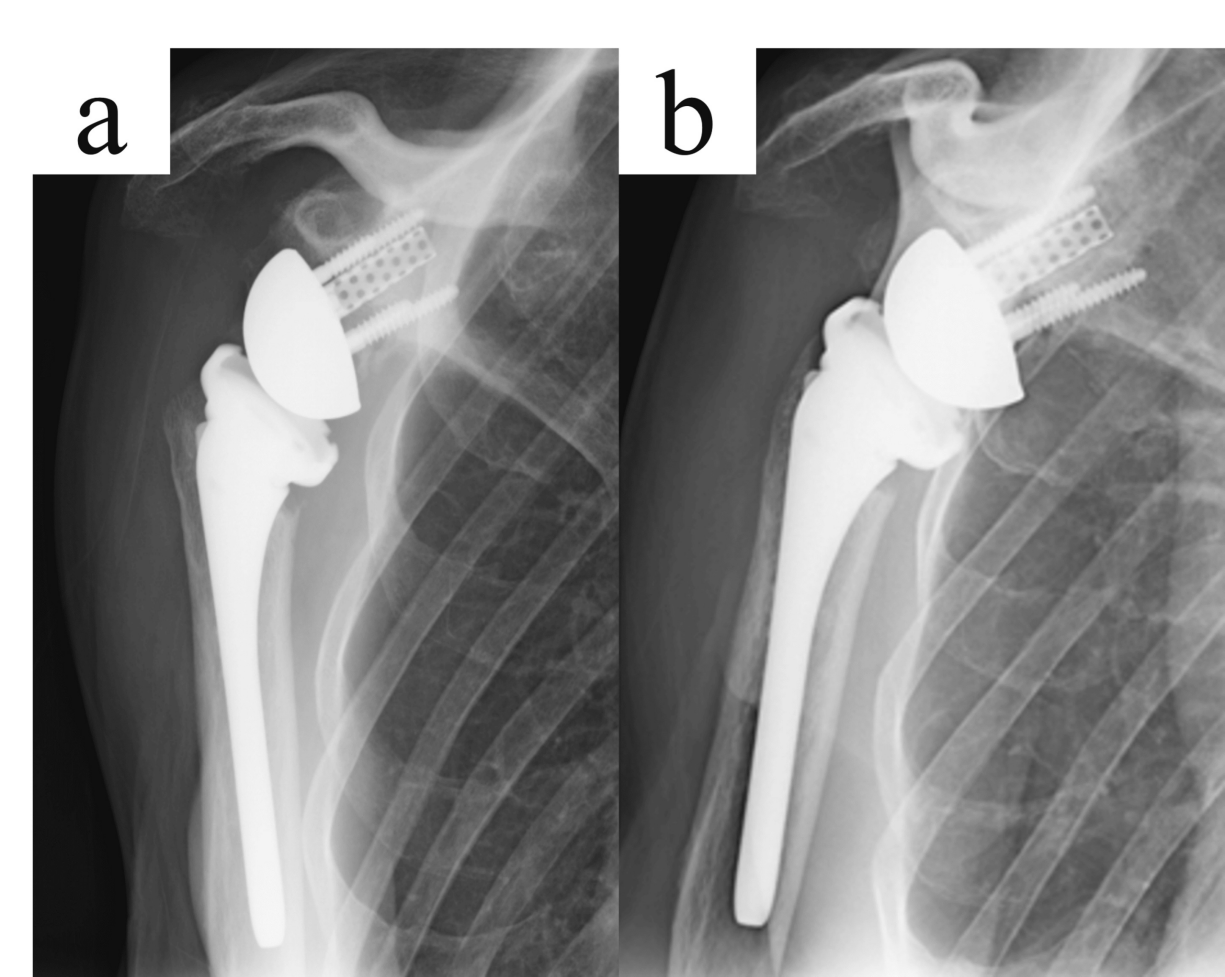
Postoperative radiographs following one-stage revision with CLAP therapy. a. Radiograph at one month postoperatively showing stable fixation without radiolucent lines. b. Radiograph at one year demonstrating maintained implant stability and no signs of reinfection or osteolysis. CLAP: continuous local antibiotic irrigation

Radiographs showed stable fixation at one month (Figure 5a) and one year (Figure 5b).

**Figure 6:**
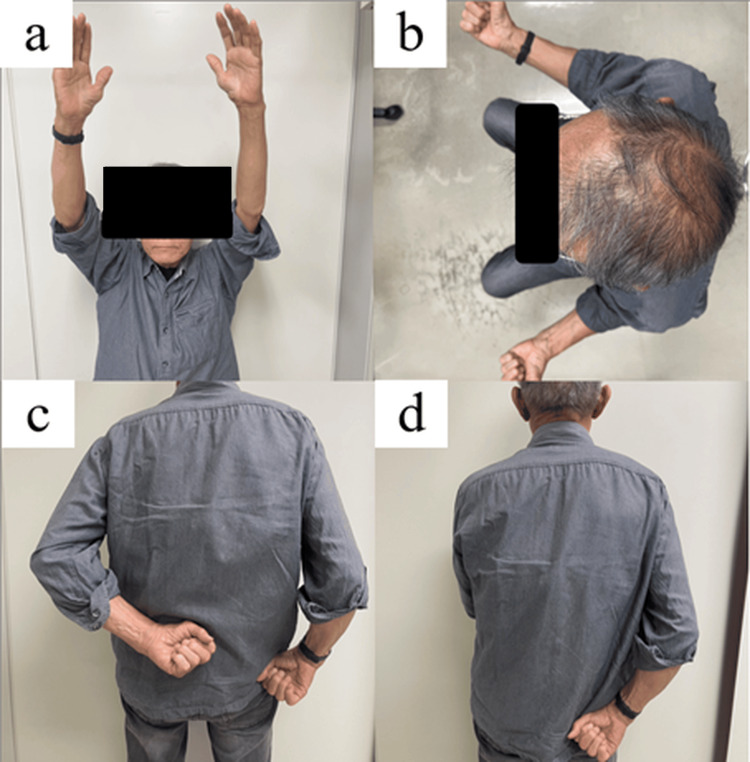
Clinical photographs showing shoulder function at one year after one-stage revision with CLAP therapy. (a) Active forward flexion: 100°. (b) Active external rotation: 10°. (c) Left internal rotation toward the L1 vertebra. (d) Right internal rotation toward the sacrum. CLAP: continuous local antibiotic irrigation

At one year, the patient was pain-free with an improved range of motion (forward flexion, 100°; abduction, 90°; external rotation, 10°; internal rotation to the sacrum) (Table 3, Figure 6). No relapse of infection was observed. Patient satisfaction was not formally quantified using a validated satisfaction instrument; however, at the final follow-up, the patient reported that the outcome was satisfactory with meaningful pain relief and improved ability to perform activities of daily living.

**Table 3:** Comparison of functional outcomes between pre-revision surgery and the final follow-up *Internal rotation shown as spinal level. JOA: Japanese Orthopaedic Association

Parameters	Pre-revision	One year after operation
Anterior elevation	100°	100°
Abduction	80°	90°
External rotation	15°	10°
Internal rotation*	Thigh	Sacrum
JOA score	38	69

The patient was pain-free and able to perform basic activities of daily living without assistance.

## Discussion

To our knowledge, based on a comprehensive search leading up to the publication of this paper, this is the first reported case of a one-stage revision for shoulder PJI caused by multidrug-resistant (MDR) organisms that was augmented by CLAP. Prior reports around the shoulder describe CLAP mainly for surgical site infection (SSI) or implant-retaining strategies (e.g., debridement, antibiotics, and implant retention (DAIR)/partial exchange) rather than one-stage exchange, and typically involve low-virulence organisms such as *Cutibacterium* spp., not multidrug-resistant (MDR) pathogens [9-11].

Historically, two-stage revision surgery has been most commonly used for chronic shoulder PJI. However, contemporary evidence and expert guidance indicate that carefully selected patients with identified pathogens, reconstructable bone/soft-tissue envelopes, and acceptable host status can achieve infection control and clinical outcomes with one-stage exchange that are comparable to two-stage approaches. This is reflected in the 2018 International Consensus Meeting (ICM) Shoulder section and in systematic reviews/meta-analyses of shoulder PJI [1,2,12]. Recent analyses of shoulder PJI report reinfection rates for one-stage revision that are at least comparable to, and in some studies modestly lower than, those observed after two-stage revision, although case-mix differences and preferential use of two-stage protocols for more virulent or resistant organisms must be considered [6,12]. Moreover, most published one-stage series predominantly involve low-virulence organisms such as *Cutibacterium acnes* and do not incorporate adjunctive continuous local antibiotic delivery; in this context, our case extends the one-stage shoulder PJI literature to an MDR gram-negative infection managed with one-stage exchange augmented by CLAP [6,9-12].

CLAP, developed and refined in Japan, enables sustained high local antibiotic concentrations at the biofilm interface while minimizing systemic exposure, which is attractive for biofilm-related and MDR infections when organisms remain susceptible to locally deliverable agents (e.g., aminoglycosides) [8,10,11]. Early clinical experiences suggest favorable safety and promising control of difficult infections in orthopedics, including knee PJI (often as DAIR+CLAP or high-concentration CLAP protocols) and shoulder SSI [9,13,14].

In the hip and knee domains, evidence supporting one-stage strategies has expanded (including clinical and cost-effectiveness), while reports specifically combining one-stage exchange with continuous local antibiotic delivery are limited; most CLAP literature centers on DAIR/implant retention [4-6,13-15]. In the shoulder, published CLAP experiences focus on SSI and DAIR/partial exchange, for example, a reverse shoulder arthroplasty PJI treated with CLAP while retaining the implant, highlighting the gap our case fills [9-11]. Conversely, in hip PJI, a one-stage THA with intra-articular antibiotic infusions illustrates the broader “one-stage + continuous local infusion” concept, aligning pharmacologic logic with our approach [16].

Beyond CLAP, local antibiotic delivery in PJI more commonly relies on antibiotic-loaded cement spacers, beads, or short-course intra-articular catheter infusions, particularly in staged revision settings [2,7,12,16]. These carriers can provide high early elution and dead-space management but are constrained by the limited set of heat-stable antibiotics that can be mixed into polymethylmethacrylate and by a characteristic decline in elution to subinhibitory levels over time, which may be suboptimal for MDR organisms [2,4,5,12]. By contrast, CLAP allows the surgeon to select one or more antibiotics according to susceptibility, set a constant perfusate concentration for a defined duration, and adjust flow rate, at the cost of requiring intact soft-tissue coverage, secure catheter fixation, and infrastructure for continuous infusion [8-10,13,14,16]. Our experience suggests that this trade-off may be acceptable in carefully selected, reconstructable shoulders with aminoglycoside-susceptible MDR organisms, but CLAP is unlikely to replace antibiotic spacers or beads in all scenarios.

In this case, MDR pathogens retained aminoglycoside susceptibility, enabling CLAP to deliver very high local drug levels while we performed meticulous debridement and immediate reimplantation, consistent with ICM-endorsed indications for one-stage exchange [1,8,10,11]. The uneventful wound course, early normalization of inflammatory markers, and durable infection control at one year without suppressive therapy suggest that, when microbiologic suitability, soft-tissue integrity, and reconstructability are present, one-stage exchange augmented by CLAP can be a rational, less burdensome option for appropriately selected patients with shoulder PJI [8-11,13,14,16].

This report has several limitations. First, its one-case nature and relatively short follow-up preclude definitive conclusions about durability and late complications (e.g., mechanical loosening or late reinfection). Second, CLAP parameters were individualized rather than standardized (drug choice, concentration, flow rate, and duration), and we did not obtain local pharmacokinetic measurements; therefore, we cannot isolate the incremental effect of CLAP from that of meticulous debridement and one-stage exchange. Third, external validity is limited: our approach presumes organism susceptibility to locally deliverable agents and adequate soft-tissue/bone conditions, and may not apply to other microbiologic or host contexts. Fourth, while our structured search supports the claim that this is the first case of one-stage revision plus CLAP for MDR shoulder PJI, we acknowledge the possibility of missed reports. Fifth, functional outcomes in this study were primarily assessed using range of motion and JOA scores. Patient-reported shoulder-specific outcome measures (e.g., American Shoulder and Elbow Surgeons Standardized Shoulder Assessment Form (ASES), Constant-Murley Score (CMS) score, Simple Shoulder Test (SST)) and standardized patient satisfaction measures were not systematically collected, limiting comparability with previous shoulder PJI literature.

Safety considerations also warrant emphasis. CLAP is designed to maximize local antibiotic exposure while limiting systemic levels, but very high local aminoglycoside concentrations raise theoretical concerns about cartilage and bone toxicity, delayed soft-tissue healing, and neurovascular irritation, and systemic absorption can still occur, with potential nephrotoxicity or ototoxicity if monitoring is inadequate [8-10,13,14]. Prolonged subinhibitory exposure could also select resistant subpopulations. Existing CLAP case series in periprosthetic knee infection and fracture-related infection have reported good infection control with low rates of clinically apparent toxicity when trough levels and renal function are regularly monitored [8-10,13,14], yet these data remain limited. In our case, no clinical signs of local or systemic toxicity were observed, but detailed pharmacokinetic sampling and formal otologic evaluation were not performed. Larger prospective studies with standardized CLAP protocols, pharmacokinetic sampling, and predefined safety endpoints are needed to clarify the risk-benefit profile of CLAP in arthroplasty-related infections.

## Conclusions

Compared with two-stage revision surgery, one-stage revision surgery for PJI shortens treatment time and maintains functional outcomes. In this case, one-stage revision surgery combined with CLAP achieved no clinical or laboratory evidence of recurrent infection at 12 months without ongoing antibiotic therapy. CLAP may represent a promising adjunctive therapy for managing complex shoulder PJIs caused by multidrug-resistant organisms.
